# Automated identification of radiotherapy treatment sites from unstructured physician notes

**DOI:** 10.1002/acm2.70558

**Published:** 2026-03-31

**Authors:** Klea Hoxha, Ricky R. Savjani, Jack Neylon, Jonathan Pham, Rojine Ariani, Dylan P. O'Connell

**Affiliations:** ^1^ Department of Radiation Oncology University of California, Los Angeles Los Angeles California USA

**Keywords:** AI in radiotherapy, large language models, workflow optimization

## Abstract

**Purpose:**

Ambiguous or incomplete documentation is a recurrent bottleneck in radiation oncology workflows, leading to inefficiencies in communication and potential treatment delays. Large language models (LLMs) pose a solution to addressing these ambiguities without added burden to clinical staff. We aim to assess the effectiveness of Meta's open‐source Llama 3.3 model in using physician consultation notes to isolate and classify anatomical treatment sites and create helpful extractive summaries for each patient.

**Methods:**

Semi‐structured interviews with five radiation therapists revealed that CT simulation orders lack the necessary details to acquire the appropriate image. A retrospective cohort of 100 patient notes was used for iterative prompt engineering. The final model was evaluated on an independent test cohort of 52 patient notes. The LLM's accuracy in identifying the treatment site was benchmarked against two human observers (a medical physicist and a physician) as well as the final delivered treatment plan (ground truth). The helpfulness and accuracy of the AI‐generated summaries were also rated by both observers on a 5‐point Likert scale.

**Results:**

Llama 3.3 achieved a weighted accuracy of 94.2% [95%CI: 89.4%–98.1%] when compared to sites isolated by either observer. When compared to the sites isolated from the retrospectively delivered plans, the model reached a weighted accuracy of 92.3% [95% CI: 87.5%–97.1%]. The model classified the anatomical sites with a weighted accuracy of 96.2% [95%CI: 87.0% –98.9%]. The AI‐generated summaries were highly rated by both observers (Observer 1: 4.96 [95%CI: 4.87–5.00] and Observer 2: 4.58 [95% CI: 4.38–4.73]).

**Conclusion:**

This pilot study provides foundational evidence that LLMs can classify data with high accuracy, achieve benchmarks comparable to human experts when isolating anatomical treatment sites, and produce clinically helpful summaries. Our results suggest that LLMs can be effectively integrated to streamline complex radiotherapy workflows in the clinic.

## 1 INTRODUCTION

The cancer burden on American society continues to grow, with an estimated 2 041 910 new cancer cases projected in the United States in 2025.^1^ Consequently, the demand for radiation therapy, along with the complexity and specificity of care, continues to rise. While novel techniques have revolutionized treatment precision and outcomes, they have also introduced additional layers of complexity into the clinical workflow. Modern radiation therapy requires careful orchestration across interdisciplinary teams, from initial consultation to treatment delivery. In this dynamic setting, clear communication is crucial to a safe and efficient clinical practice.

Aside from the inherent demands that clinical staff face when preparing to coordinate radiation treatments, documentation accuracy remains a challenging yet addressable workflow obstruction.^2^, ^3^ While many institutions have adopted structured formats to standardize communication, studies show that data entry failures and ambiguities remain a significant source of error in modern workflows. A recent publication by Cao et al. points out the inherent variability in CT simulation orders at their institution.^4^ Riegel et al. report that wrong target dose, miscommunication on treatment strategy, incorrect laterality, and omitted treatment devices are some frequently reported failure modes in their institutional Radiation Oncology Incident Learning System (RO‐ILS) database.^5^ At the national level, Ezell et al. analyzed 396 high‐priority events and found that 25% (99 of 396) were categorized as “problematic plan approved for treatment,” 65% of which were attributed to incorrect target definition or dosing patterns.^6^ Collectively, these studies show that structured documentation does not eliminate communication errors and ambiguities that can contribute to workflow inefficiencies and delays in care. Previous work has shown that long delays at the start of a radiation treatment can lead to decreased tumor control and increased levels of distress, anxiety, and depression in patients.^7^, ^8^, ^9^, ^10^

Large language models (LLMs) have seen an increase in popularity due to their strong performance across academic and professional domains. These models have transformed natural language processing (NLP), and their application in various medical settings is being evaluated.^11^, ^12^ A recent scoping review shows that a majority of LLM‐based applications in oncology from 2021 to 2024 explore general medical question–answering tasks and diagnostic reasoning.^13^ A second review study shows that prior work on LLM‐based clinical text summarization has largely targeted radiology reports, progress notes, patient forms, or structured clinical orders.^4^, ^14^ To our knowledge, there is a lack of research aimed at the extraction of detailed planning parameters from unstructured consultation notes for downstream end‐users.

Although LLMs show great promise in many fields, their deployment must be balanced against computational demands and compared to traditional rule‐based NLP methods. Despite the long history of NLP research in the clinical sphere, progress in clinical text processing has been slow due to barriers such as limited shared data, insufficient annotated datasets for training and benchmarking, heterogeneous annotation conventions, and poor generalizability. Despite the long history of NLP research in the clinical sphere, progress in clinical text processing has been slow due to barriers such as limited shared data, insufficient annotated datasets for training and benchmarking, heterogeneous annotation conventions, and poor generalizability. Scalability also becomes difficult when tools are over‐fitted to specific applications and require expert input to apply to similar problems.^15^, ^16^, ^17^, ^18^ Clinical narrative necessitates the use of annotations and shorthand, which often vary not only between institutions but also between physicians. This proves problematic for general text NLP models. Additionally, despite recent advances in temporal extraction, distinguishing past conditions from current or future ones remains problematic for NLP methods.^19^, ^20^ As the next step in NLP evolution, we argue that generalized LLMs have the capacity to address many of the mentioned shortcomings using only prompt engineering techniques, which are more streamlined than traditional NLP rule‐based methods (Table S1, Supplementary Materials).

This pilot study aims to explore the capabilities of Meta's open‐source Llama 3.3 LLM to process unstructured radiation oncologist consultation notes prior to CT simulation. The model is prompted to (1) isolate and classify the correct anatomical treatment site, and (2) provide a standardized extractive summary that includes relevant clinical context (e.g., prior imaging, implants, surgical history). In addition to bridging the research gap identified above, our model is designed to address an internal workflow obstruction and support the preplanning and CT simulation workflows for medical physicists, dosimetrists, and radiation therapists. This technique uses existing documentation to improve efficiency without added cognitive burdens on clinical staff and physicians, who already face documentation burnout.^21^

## 2 METHODS

### 2.1 Proposed clinical workflow

In our current workflow, physician intent is documented across two complementary sources: the physician consultation notes (text‐heavy narrative describing disease extent, history, imaging findings, and treatment details) and the CT simulation order (structured document with free‐text fields for treatment site and simulation instructions). Although the CT simulation order streamlines communication between physicians and downstream users, treatment‐site entries may lack anatomical granularity in routine practice (e.g., “lung” rather than “left lower lobe”). Planners (physicists/dosimetrists) routinely review both documents prior to planning to confirm target details and identify relevant imaging findings and dates for fusion and planning context. In contrast, therapists have limited time at the scanner and primarily rely on the CT simulation order, making it impractical to search long narrative notes during simulation. A de‐identified example illustrating the documentation gap between the detailed consultation note versus coarse treatment‐site entries in the CT simulation order is provided in Table S2, Supplementary Materials.

**FIGURE 1:**
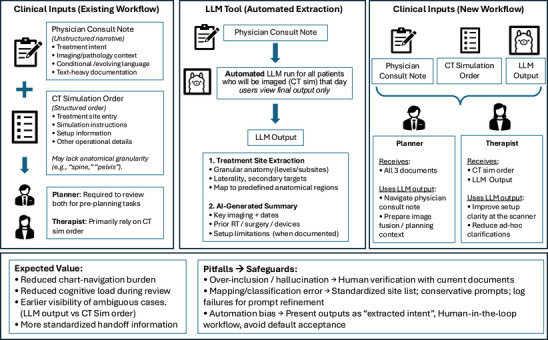
Intended clinical use case of the LLM tool. The model runs after consultation note finalization and produces a structured treatment‐site extraction and consult‐note‐derived summary. Outputs are provided to planners and therapists prior to CT simulation and planning to support verification and clarify documented intent.

The proposed LLM tool is designed to execute on institutional GPUs after consultation note finalization without user interaction. It generates two outputs displayed in the clinical dashboard: (1) treatment‐site extraction and (2) a consult‐note‐derived AI summary. These outputs are intended to be available to planners and therapists prior to CT simulation and planning to improve the readability of documented intent and reduce manual information extraction from narrative notes. Because the LLM outputs are derived from the consultation note, discrepancies between the CT simulation order and the LLM output can serve as a practical cue for closer review and clarification. The tool is not intended to replace physician documentation or required chart review, and it cannot infer missing documentation or fix incorrect physician intent; thus, human verification remains required. Figure 1 illustrates the intended clinical use case and integration point in the workflow.

### 2.2 Radiation therapist interviews

This study was granted an exemption from formal review by our Institutional Review Board (IRB). Prior to study design, informal qualitative feedback from the planning and therapy teams highlighted inconsistent simulation order clarity as a source of operational delay. To establish a baseline for CT simulation order quality at our institution, five radiation therapists participated in semi‐structured interviews. The interviews covered each therapist's experience with the current level of detail in CT simulation order, examples of disrupted simulation and planning workflows, the most common issues for contacting physicians, and how these issues were typically resolved. The interviews were recorded and transcribed by the research team, with all personal identifiers removed. The most frequently mentioned sources of presimulation delays were categorized.

### 2.3 Large language model setup

Meta's open‐source 70B instruction‐tuned Llama 3.3 model, accessed via Hugging Face transformers, was tested for its accuracy on identifying and classifying the correct anatomical treatment site(s) from radiation oncologists’ consultation notes. Inference was performed on two 80GB A100 GPUs (max tokens: 1024) with fixed decoding parameters. Temperature was set to 0.5 and nucleus sampling (top‐p) to 0.9 to balance output stability with sufficient variability while limiting low‐probability generations.^22^, ^23^ The model ran entirely on institutional GPUs, with access restricted to authenticated users, which ensures that no patient data were transmitted outside the institutional firewall.

**FIGURE 2:**
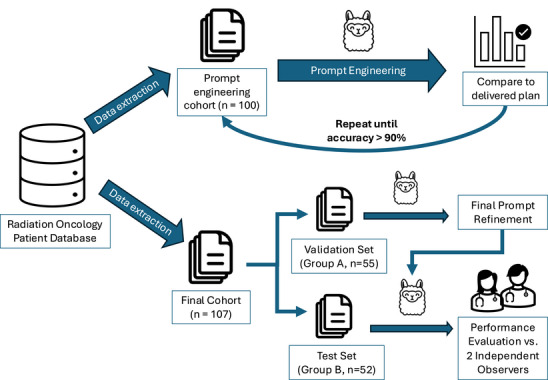
Study workflow outlining the development and validation of the LLM tool. The initial prompt engineering phase was performed on a cohort of 100 patient cases, followed by a validation step using 55 patient cases. Finally, the model's performance was evaluated on an independent test cohort (*n* = 52) against two human reviewers.

Two retrospective patient cohorts were selected from our institution's scheduling software. The first cohort consisted of the most recent consultation notes from 100 consecutive patients receiving radiation therapy during the study period. This initial cohort was used for iterative prompt engineering and performance optimization. The second cohort was selected via systematic sampling. A list of patients who were treated during the study was generated and sorted by MRN. The first 107 patients were selected, and the cohort was split into Groups A (*n* = 55) and B (*n* = 52). The outputs for Group A were evaluated by a medical physicist, and final prompt revisions were performed. Group B was used to report on the model's final performance and how it compares to two separate human reviewers. Figure 2 shows a detailed schematic of the study's workflow.

### 2.4 Site isolation prompt engineering

The initial set of prompts had a simple instructional setup. A role‐playing system prompt was used to instruct the model to adopt the role of a radiation therapist: “*You are a radiation therapist that takes CT images of the anatomical site(s) that will be treated*.” The physician's consult notes are text‐heavy and often contain more information than needed for this task. A multistep prompt strategy was used to isolate the most relevant sections from the text‐heavy consultation note (Prompt 1), then identify the treatment site(s) (Prompt 2), and finally classify the site(s) based on a standardized list of anatomical sites and regions provided in Table 1 (Prompts 3 and 4). The prompt engineering process included chain‐of‐thought, role‐playing, and constraint‐based techniques. These methods allowed the LLM to break down the task of isolating and classifying the treatment site into smaller, multistep subtasks and retain contextual history for each step.

**TABLE 1:** Distribution of training and testing datasets based on anatomical regions.

Anatomical region	Prompt engineering cohort (*n* = 100)	Validation set Group A (*n* = 55)	Test set Group B (*n* = 52)
	Count	Count	Count
Head and neck	40	15	11
Thorax	45	11	10
Abdomen	16	4	6
Pelvis	26	22	23
Extremities	6	3	4
Other body	0	0	1
**Total** ^a^	**133**	**55**	**55**

*Note*: Each dataset used for the study is described based on use and how many counts of each anatomical region were identified.

^a^ Counts per site may exceed the number of patients (*n*), as individual cases could include multiple treatment sites.

### 2.5 AI summary prompt engineering

A similar methodology was used to obtain a short AI‐generated summary that extracts patient information helpful to the planners and radiation therapists. The task was broken down into three prompts. Similar to the treatment site isolation task, the first prompt instructed the model to isolate the section of the physician's note with the most information on the upcoming treatment plan. Then the LLM was prompted to generate a summary including diagnosis, treatment site(s), imaging information and dates, surgeries and other procedures (hydrogel insertion/fiducial placements), radiation treatment history, concurrent treatments, mobility and setup limitations, pacemakers/cardiac devices, departmental trials, and organs at risk. The final prompt instructed the model to create a bullet point list from the summary for improved readability.

### 2.6 Iterative refinement

Notes from the initial 100‐patient cohort were used for iterative prompt engineering. Prompts were refined over 20 cycles against the ground truth until the model achieved a weighted accuracy above 90% on a three‐tier rating scale (1 = correct, 0.5 = partially correct, 0 = incorrect). The prompts were refined to address issues such as incorrect identification, classification, hallucinations, and excessive verbiage. Adjustments included the use of f‐strings, modifications of keywords, increased constraint specificity, providing example outputs, as well as explanations of specific terminology and anatomical locations.

The second patient cohort was split into two Groups A (*n* = 55) and B (*n* = 52). Group A was used to compare model performance to that of a medical physicist and obtain feedback on the AI summaries. Prompt revisions were performed, and the final optimized prompts are provided in Appendix A. Group B was used to report on the model's final performance when compared to that of a medical physicist (Observer 1) and radiation oncologist (Observer 2). Selecting these roles as reviewers ensured that we captured diverse perspectives from two distinct ends of the workflow, note authors (physician) and downstream users (physicists). This allowed the evaluation to address both medical accuracy and technical utility. Each observer independently isolated the site(s) and rated the AI summaries. Table 1 shows the dataset distribution and counts for each treatment site.

### 2.7 Statistical analysis

The weighted accuracy of the AI‐generated outputs was assessed by comparing them to two different reference standards. The first comparison assessed LLM performance against the ground truth, established retrospectively by reviewing dose distribution and structure overlays on the delivered plans. This allows for an evaluation of the model's ability to infer the true treated site, independent of document quality. The second comparison evaluated how the LLM performed relative to what site a human reviewer can isolate from the physicians’ notes. While rare, discrepancies in treatment sites between the consultation and treatment day may occur, mainly due to diagnostic imaging or other procedures not being available at the time of consult. Because of these discrepancies and because the model can only operate on what is documented in the source text, LLM outputs were compared to those of human reviewers in addition to the true treated site.

Site isolation was scored on a three‐tier scale: 1 point for correct identification, 0.5 points for partially correct identification (missing secondary sites, incorrect laterality, node inclusion), and 0 points for incorrect identification. Mean accuracy scores were calculated and reported as percentages across all evaluation standards. The three‐tier rating scale could potentially mask clinically significant errors, which is why a more granular error analysis was performed. Each LLM failure instance was categorized and compared to the failures of human observers. Additionally, both observers were asked to rate each LLM failure output on a Likert scale of 1–5 based on clinical significance. Site classification was evaluated via a binary accuracy (correct/incorrect).

A three‐way percent agreement analysis was performed between the LLM and the human observers. Uncertainty of the primary metrics was calculated using 95% confidence intervals (CI). For all binomial metrics, confidence intervals were calculated using the Wilson score interval method. For weighted accuracy scores (3‐point rating scale), CIs were calculated using a bootstrap method with 10 000 resamples. The purpose of the AI‐generated summary is to provide helpful information to the therapists and planners with the intent of facilitating the workflow. The summaries were graded by two different observers, a medical physicist and a radiation oncologist. Each summary was ranked on a scale of 1–5 based on accuracy and helpfulness to the planning process. Interobserver variability for the Likert‐scale ratings was assessed using weighted Cohen's kappa with quadratic weights, and 95% CI for the mean scores were calculated using the bootstrapping method described above. All performance metrics are reported at the cohort level, where each case contributes equally. Region‐stratified performance was not performed due to limited sample sizes in some regions (Table 1).

## 3 RESULTS

Semi‐structured interviews with radiation therapists confirmed that ambiguous treatment site details in the CT simulation orders were one of the most frequent disruptors to the clinical workflow, often requiring physician clarification and leading to potential delays. Table 2 lists the CT simulation workflow deficiencies and ambiguities as identified by the five therapists interviewed, the frequency of mention, and examples.

**TABLE 2:** CT simulation document ambiguities.

CT sim issues	Mention frequency	Example
Incorrect/Ambiguous site	5	Spine, instead of T2‐T5
Missing scan parameters	4	Not clarified where to begin/end scan
Immobilization device	2	Incorrect mask, or not included
Patient setup instructions/limitations	5	Arms up/down; patient unable to lay down
Treatment technique	2	Not specified

*Note*: CT simulation documented issues reported by five therapists during their semistructured interviews. The frequency of mention along with an example are added for each issue identified by the therapists.

### 3.1 LLM and human performance compared to ground truth

**FIGURE 3:**
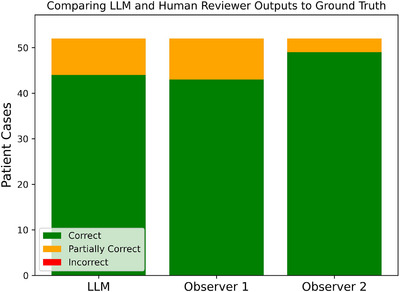
Comparison of Llama 3.3, Observer 1, and Observer 2 outputs to the treated anatomical site specified in the delivered plan documents. The graph shows no fully incorrect instances by any reviewer. The LLM identified 44 sites correctly, and eight sites partially correct, while Observer 1 identified 43 sites correctly, and nine sites partially correct, and Observer 2 with 49 correct sites and three partially correct sites.

The accuracy of the LLM and two human observers in identifying the anatomical treatment sites was retrospectively evaluated by comparing their outputs against the delivered treatment plan (ground truth). Out of 52 patient cases, the LLM identified 44 sites correctly, and eight sites partially correct, with a weighted accuracy of 92.3% [95% CI: 87.5%–97.1%]. For comparison, Observer 1 achieved an overall accuracy of 91.3% [95% CI: 85.6%–96.2%], while Observer 2 achieved an overall accuracy of 97.1% [95% CI: 93.3%–100.0%]. No fully incorrect sites were reported by the LLM or either observer. The comparative performance of the LLM and two observers is detailed in Figure 3. The LLM classified 96.2% [95% CI: 87.0%–98.9%] of the sites correctly based on a standardized list of anatomical sites.

### 3.2 Disagreement analysis

To provide a more clinically meaningful evaluation of the partial error instances, a detailed qualitative analysis was performed on all eight disagreement instances between the LLM and the ground truth (Table 3). The two most frequent error types were *incorrect site granularity* (*n* = 3) and *ambiguity of nodal involvement* (*n* = 3). *Incorrect site granularity* occurred when the model misinterpreted clinical context about the upcoming treatment plan and overestimated the number of sites that were treated. *Ambiguity of nodal involvement* occurred when the model failed to correctly determine whether local nodes were part of the treatment plan. The final failure mode, *insufficient information in source text* (*n* = 2), occurred in cases where the information in the physician's note had changed or was not sufficient to infer the standard of care. Additional error analysis of each failure mode was performed to show the exact language from the source text that led to the disagreement from the ground truth [Appendix B].

**TABLE 3:** Disagreement analysis table.

Disagreement category	LLM count	Description	Example
Incorrect Site Granularity	3	The model extracts real entities from the physician's notes but misinterprets context assigning additional anatomical sites to the treatment site list.	Correct site: para‐aortic node LLM site: vaginal apex, para‐aortic node
Ambiguity of Nodal Involvement	3	The model fails to correctly identify presence or absence of lymph nodes from physician's note, a task difficult for human reviewers alike.	Correct site: prostate LLM site: prostate with nodes
Insufficient information in source text	2	The treatment sites cannot be fully identified from the note or changes from day of consult to day of treatment.	Correct site: right tonsil, bilateral neck LLM site: right tonsillar pillar, right cervical node mass, right submandibular space

*Note*: The table shown categorizes all partial disagreements in three groups. The most frequent categories are Incorrect Site Granularity and Ambiguity of Nodal Involvement.

In addition to categorizing and analyzing the outputs, both human observers were asked to rate each of the LLM disagreement instances on a 5‐point Likert severity scale. The reviewers assigned a rating of “5” when the LLM output included entirely unrelated sites from the corresponding ground truths. Observer 1 reported an average of 4.12, while Observer 2 reported an average of 3.75. Both observers agreed on 100% of the severe mismatches that would be too incorrect for safe use in the clinic (Likert = 5). Table 4 shows all disagreement instances along with the ground truth and each observer's ratings.

**TABLE 4:** Disagreement instances Likert rating.

LLM output	Ground truth	Observer 1 rating	Observer 2 rating
Prostate and nodes	Prostate	3	2
**Para‐aortic nodes, vaginal apex**	**Para‐aortic**	**5**	**5**
Prostate bed, nodes	Prostate bed	3	2
**RLL, LUL, right lung, LLL**	**RLL, RUL**	**5**	**5**
**Prostate, L3, sacrum**	**Sacrum, L3**	**5**	**5**
**Right frontal convexity, medulla**	**Right frontal convexity**	**5**	**5**
Rectal/Anal mass	Rectum and nodes	3	3
Right tonsillar pillar, right cervical node mass, right submandibular space	Right tonsil, bilateral neck	4	3

*Note*: The table below shows each LLM output failure, the ground truth, and the Likert ratings (5 = *Severe Mismatch*) from Observer 1 and Observer 2.

### 3.3 LLM performance compared to human observers

**FIGURE 4:**
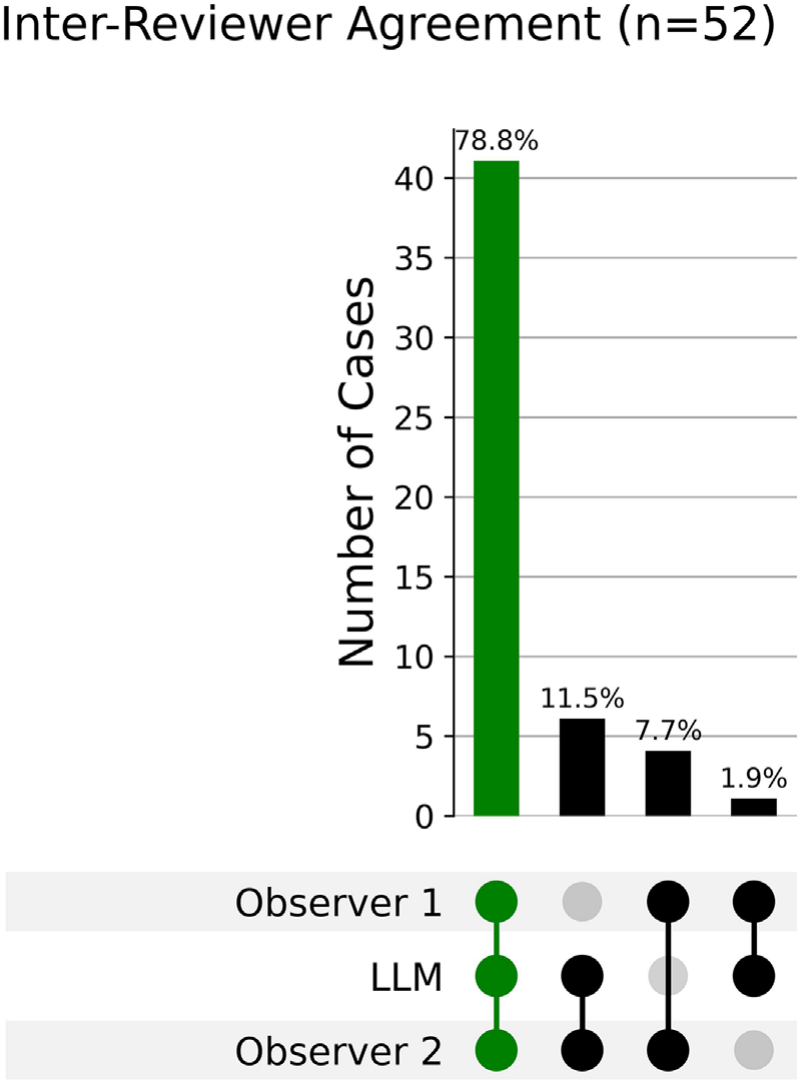
UpSet plot of agreement between LLM and two observers.

To evaluate performance relative to human interpretation, the outputs were compared to the treatment sites identified by two human observers. Accuracy was once again graded on a three‐tier scale. Overall, Llama 3.3 achieved an accuracy of 94.2% (95% CI: 89.4%–98.1%) when compared to the sites isolated by either human observer. Agreement across the LLM, Observer 1, and Observer 2 was also assessed using a binary (agree/disagree) metric and visualized using an UpSet plot (Figure 4). In the plot, agreement categories are mutually exclusive. The three‐way agreement represents cases where all three reviewers selected the same treatment site: 78.8% (41/52; 95% CI: 66.0%–87.8%)

UpSet plot showing mutually exclusive agreement patterns across Observer 1, the LLM, and Observer 2. Bars indicate the number (and percent) of cases where the marked reviewers selected the same treatment site. Leftmost bar: full three‐way agreement (41/52, 78.8%). Remaining bars: exclusive pairwise agreement where exactly two reviewers agree and the third differs (LLM + Observer 2: 6/52; Observer 1 + Observer 2: 4/52; Observer 1 + LLM: 1/52). Bars sum to 100%, no cases showed three distinct sites.

### 3.4 AI summary evaluation

**FIGURE 5:**
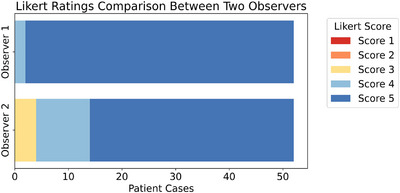
Likert rating comparison chart. Stacked bar chart comparing the distribution of Likert scale ratings for AI summary helpfulness between two independent observers for 52 patient cases. Scores range from 1 (*least helpful*) to 5 (*most helpful*). Average scores Likert scores of each observer were 4.96 for Observer 1 and 4.58 for Observer 2.

AI summaries were generated to provide an easily accessible source of important information to radiation treatment planners and therapists prior to CT simulation and planning. Two independent observers were asked to read the physician's consultation notes and AI summaries for each patient and rate them from 1 to 5 (*Worst* to *Best*) based on helpfulness and accuracy. The average scores from each observer were calculated to be 4.96 (95% CI: 4.87–5.00) for Observer 1 and 4.58 (95% CI: 4.38–4.73) for Observer 2. Figure 5 shows a stacked bar chart comparing the distribution of the Likert scale rating for the AI‐generated summaries for each observer. The raw percent agreement between the two observers was high at 73.1%. To account for agreement due to chance, a weighted Cohen's Kappa was calculated. As a result of the highly skewed distribution of ratings towards the maximum scores, the calculated kappa value was low (*κ* = 0.13), indicating only slight agreement beyond chance. This is a known statistical artifact encountered in cases with low rating variability, known as the Kappa Paradox.^24^ Input/Output examples for the site identification/classification and AI‐generated summaries are shown in Table 5.

**TABLE 5:** LLM input/output example.

Partial input (part of physican's consultation note)	Output
…Recent imaging: DD/MM/YYYY MR neck Objective: LMP DD/MM/YYYY (exact date) Restricted in physically strenuous activity but ambulatory and able to carry out work of a light or sedentary nature, e.g., light housework, office work. Appearance: normal appearance. She is normal weight. Comments: Indurated endophytic mass in right lateral tongue, no exophytic component, site ulceration Musculoskeletal: Cervical back: normal range of motion. Neurological: Mental status: She is alert. Assessment: Patient is a XX y.o. female with LEFT oral tongue SCC LEFT partial glossectomy on DD/MM/YYYY, and pT1N0 RIGHT floor of mouth SCC RIGHT composite resection of mandible and soft tissue with right MRND on DD/MM/YYYY, with subsequent LEFT floor of mouth moderately differentiated SCC 2 cycles neoadjuvant carbo/taxol with minimal response, composite resection LEFT mandible, hemi‐tongue, and surrounding tissues + left MRND on DD/MM/YYYY (ypT4aN0cM0, 5.0 cm primary involving the mandible, left SMG, and skeletal muscle, +extensive PNI, neg margins, 0/32 LNs involved), adjuvant RT completed on DD/MM/YYYY. Had local recurrence in her neck re‐irradiation with SBRT completed on DD/MM/YYYY. Now biopsy proven well‐differentiated SCC of right posterior lateral tongue. She continues immunotherapy and will proceed to re‐RT. Plan/Recommendation: proceed to re‐RT to the new right tongue lesion. Discussed traditional every other day treatment schedule versus a more PULS approach of every other week. The latter approach is likely to minimize risk of toxicity and may synergize better with concurrent IO. —CT Sim next available, with treatment in LOCATION on STX…	**Identified site**: Right tounge. **Classification**: Oral cavity, head
	**AI summary**: The diagnosis is squamous cell carcinoma of the right posterior lateral tongue. Anatomical site to be treated: right posterior lateral tongue Relevant imaging: – PET/CT (DD/MM/YYYY) – MRI neck (DD/MM/YYYY) – CT face/neck with contrast (DD/MM/YYYY) – CT neck with contrast (DD/MM/YYYY) – CT chest without contrast (DD/MM/YYYY) Open Imaging orders: none mentioned. Prior radiation history: The patient has a history of adjuvant radiation therapy completed on DD/MM/YYYY and re‐irradiation with SBRT completed on DD/MM/YYYY. No pacemaker or cardiac device. Concomitant treatments: The patient is receiving concurrent immunotherapy. The patient is post‐operative: undergone multiple surgeries, including left partial glossectomy, bilateral mandible resection, and modified radical neck dissections. Organs at risk: spinal cord, salivary glands, oral cavity, mandible. Neurosurgery required: no. Neuro/Coplanning: none. No reported mobility issues. Department trial involvement: no.

*Note*: The table below shows an excerpt of the consultation note (input) and the outputs for both the site classification/isolation and AI‐generated summaries.

## 4 DISCUSSION

The development of this tool was directly motivated by an internal workflow bottleneck where recurring clinical feedback from planners and therapists identified treatment‐site ambiguity as a workflow disruptor. Semi‐structured therapist interviews confirmed the need for a tool that addresses information gaps prior to simulation, particularly treatment‐site ambiguities and incomplete setup instructions (Table 2). Our study shows that the developed model can mitigate these bottlenecks by (1) extracting and classifying detailed anatomical treatment sites from physician consultation notes and (2) generating a concise, standardized summary that surfaces key clinical context to support CT simulation and preplanning. In practice, these structured consult‐note‐derived outputs are intended to be available to therapists and planners prior to simulation and planning. This way, therapists gain access to relevant consult‐note context that is typically impractical to retrieve at the scanner, while the planner's review can shift from manual information extraction within a text‐heavy narrative toward verification and spot‐checking of model outputs. The summary also captures setup‐relevant limitations (e.g., mobility constraints) when documented in the narrative. Thus, the structured summaries, along with the detailed treatment site extraction, address two of the most frequently mentioned bottlenecks from the interview phase. Other components, such as scan parameters, immobilization device selection, and treatment technique, remain managed through existing clinical documentation (CT simulation orders and consultation notes).

### 4.1 Large language model performance analysis

Quantitatively, our model demonstrated robust performance with a weighted accuracy of 92.3%, comparable to that of a medical physicist (Observer 1) and a radiation oncologist (Observer 2): 91.3% and 97.1%, respectively. While this metric established a baseline performance overview, it did not distinguish between error types in terms of clinical significance: Missing laterality and nodal involvement errors were both scored the same. Therefore, a Likert‐scale analysis of the disagreement instances was conducted to better assess the model's safety and performance (Table 3). Inspection of the eight disagreement instances revealed high average severity scores of 4.12 and 3.75 by Observers 1 and 2, respectively (Table 4), implying substantial deviation from ground truth. However, disagreement cases were not uniformly “model‐only” failures. Four out of the eight model failure instances overlapped with errors made by at least one human reviewer (Table 6). This notable error overlap suggests that the high severity scores are, at least partially, due to the inherent difficulty of interpreting consultation notes. In this context, interpretation difficulty arises from evolving treatment intent (e.g., “may benefit from RT” pending results) and documentation that describes multiple lesions (treated vs. discussed incidental findings). Additional challenges include clinical shorthand and cases where imaging/pathology results are not yet available at the time of consultation or are embedded in dense paragraphs, as shown in Tables 5 and S2, Supplementary Materials.

**TABLE 6:** Shared partial failure instances.

Treated site	Observer 1	Observer 2	LLM	Severity score
Sacrum, L3	Prostate, L3, left sacrum	Prostate, L3, sacrum	Prostate, L3 vertebral body, left sacrum	Observer 1: 5 Observer 2: 5
Rectal mass and nodes	Rectum	Rectum	Rectal/Anal mass	Observer 1: 3 Observer 2: 3
Right frontal convexity	Right frontal convexity, left medulla	Right frontal convexity	Right frontal convexity, left medulla	Observer 1: 5 Observer 2: 5
Bilateral neck, right tonsil	Right neck	Right tonsil	Right tonsillar pillar, right cervical node mass, right submandibular space	Observer 1: 4 Observer 2: 3

*Note*: The table shows all instances of partially incorrect site identification from the LLM and both observers.

Although end‐user validation by therapists remains the most direct assessment of therapist‐facing ambiguity at CT simulation, our selection of a physician and a physicist provides a rigorous downstream clinical‐actionability and safety evaluation. In our current workflow, definitive treatment intent is determined by the radiation oncologist and documented in the consultation note and CT simulation order. Planners (medical physicist/dosimetrist) review both documents prior to planning. By selecting two complementary reviewers, we aim to encompass whether the extracted site faithfully reflects the physician intent and oncologic context (as defined by physician), and whether the output contains sufficient anatomical specificity to proceed with planning (physicist). Therefore, outputs flagged as a “severe mismatch” (Likert = 5) reflect cases where clarification would be warranted. Notably, the two observers had a 100% agreement on the four cases rated as severe mismatches (Table 4). Of these, two cases (50%) reflected errors also made by a human expert (Table 6), reinforcing that model performance is intrinsically linked to source text. Overall, the high agreement on which cases are severely mismatched and the robust model performance when compared to ground truth support the conclusion that the LLM tends to reduce ambiguity in standard cases.

The first failure in Table 6 is an example of a plan change from consultation to simulation due to prostate biopsy results being negative for active disease. While instances where the treatment site changes from the day of consultation are rare, they emphasize that model outputs are bounded by the source text available at consultation, and that comparison to human interpretation is a required benchmark. We also observed instances of model self‐correction where the LLM initially reported an incorrect secondary site but, when prompted to justify its reasoning, the model provided only the correct site in the final output [Appendix C]. In practice, this type of verification prompt can be implemented as an automated prompting step within the multiprompting pipeline (triggered without user interaction) and presented to downstream users as the final, reviewable output. Similar findings have been reported previously, where prompting techniques that elicit a model's reasoning can reduce errors and improve overall performance.^25^, ^26^

Qualitative review of the eight disagreement cases (Table 4), [Appendix B], revealed recurring error patterns. Three of eight partial failures were due to the model incorrectly assigning nodal involvement. One of these cases (rectal mass and nodes) was also miscategorized by the human reviewers. The remaining two cases, both prostates, were unique failures of the model. It is plausible that these instances were direct artifacts of our prompt engineering strategy, which was designed to extract definitive statements on nodal status: “*When the site to be treated is prostate or prostate bed, report pelvic lymph nodes in the area ONLY IF they are being treated”* [Appendix A]. This structure creates a forced‐choice scenario for the model without providing a way to report uncertainty. Future work will explore the incorporation of different prompting language and model self‐correction strategies.

In four out of eight partial failures, the model reported additional anatomical treatment sites that were explicitly mentioned in the note but not treated (the vaginal apex in a para‐aortic recurrence, additional PET‐avid lung lesions, prostate for the sacral/L3 metastases, and a second extra‐axial lesion near the medulla). Interestingly, these outputs were rated as most severely mismatched by both observers (Table 4). Because these added sites were traceable to the source text, these failures are more consistent with extraction from ambiguous documentation than random hallucination. In practice, disagreements between consult‐note‐derived LLM outputs and manually completed CT simulation orders could serve as a useful signal for closer inspection by human reviewers, underscoring the importance of maintaining a human‐in‐the‐loop workflow when deploying AI in the clinic. We believe that LLM behavior patterns suggest that the model could provide implicit flagging of documentation ambiguities. However, this potential application should be validated using explicit prompting language, larger cohorts, and additional human reviewers.

### 4.2 Large language models as clinical tools

Agreement analysis (Figure 4) shows that the developed model has a higher exclusive agreement with the radiation oncologist (Observer 2: 11.5%, *n* = 6) than the medical physicist (Observer 1: 1.9%, *n* = 1). Observer 2 reported relying on clinical expertise to interpret ambiguous notes. The LLM's stronger agreement with the physician suggests that the model uses textual analysis and cues in a way that parallels clinician decision patterns. This implies that the model may be capturing the narrative logic of the consultation note, for example, cases with abnormal findings involving node(s) on imaging or pathology reports, as opposed to the physicist who may prioritize direct order definitions. The high three‐way consensus of 78.8% indicates that the extraction task is reproducible and objective for most standard cases. Consensus breakdown occurs in cases that require interpretation or more robust documentation, emphasizing the importance of human oversight for clinical implementation of AI models. Beyond its utility in identifying the correct treatment site, the model's high classification accuracy (96.1%) further demonstrates its capacity as a powerful tool for automating and standardizing clinical data.

The LLM‐generated summaries for each patient were rated as predominantly helpful by each independent observer (Figure 5). The high Likert scores, 4.96 (95% CI: 4.87–5.00) for Observer 1 and 4.58 (95% CI: 4.38–4.73) for Observer 2, show the LLMs’ potential to help facilitate clinical workflow by providing straightforward access to critical information for radiation planners and therapists. A study by Van Veen et al. demonstrated that LLMs can outperform human experts in clinical text summarization and are often preferred over human expert summaries. The study implies that clinical integration of LLMs could alleviate documentation burden and lead to more personalized healthcare.^25^ Our findings confirm the robustness of LLM‐generated summaries and their value in the clinical sphere.

Observer 1 ratings showed less variability, with 96% of the scores being a “5.” In contrast, Observer 2 ratings were distributed across scores of “3” (7.7%), “4” (26.9%), and “5” (65.4%). The high Likert rating percent agreement (73.1%), along with the skewed distribution, indicates a strong consensus between the observers that the AI summaries were overall helpful. The low Kappa value is a product of the sensitivity of the statistical tool to unbalanced and skewed ratings, known as the Kappa Paradox.^24^ The difference in rating distribution likely stems from role‐specific perspectives. While the medical physicist's (Observer 1) focus might gear towards the technical utility of the AI summaries for the planning process, the physician's (Observer 2) focus might include a nuanced clinical perspective of the patient's condition.Neither observer reported any clinically detrimental errors in the AI summaries.

### 4.3 Study limitations

While our results suggest LLMs show great promise as clinical tools, several limitations must be addressed. First, generalizability is limited by setting, sample size, and distribution. All the data used for this study were obtained from a single institution, which can introduce documentation bias since templates, abbreviations, and clinical shorthand can vary between institutions. The sample size used to analyze prompt and model performance was 52 cases, with two human reviewers recruited for result comparison and rating. In addition, some anatomical regions were underrepresented (Table 1), so region‐specific performance cannot be reliably estimated. While sufficient to show promise in a pilot study, stratified multi‐institutional cohorts and additional reviewers are needed to better characterize performance variability across disease sites and documentation styles.

Second, end‐user and implementation outcomes were not directly evaluated. Our reviewer selection ensures a diverse perspective between opposing ends of the clinical workflow, from the physician to the technical end‐user, the physicist. However, we did not formally evaluate the LLM outputs with the radiation therapists, nor did we prospectively measure workflow impact metrics such as planner time burden, chart‐navigation burden, or downstream simulation delays. As a result, our findings demonstrate retrospective model performance rather than quantified improvements in clinical efficiency or safety outcomes.

Third, model behavior is bounded by documentation quality and can introduce failure modes that require safeguards. Although prompt engineering improved the model's overall performance, a systemic error was observed where the LLM incorrectly classified systemic therapies as “chemotherapy.” We were also able to identify instances where initially, the model correctly isolated the treatment site in a plan summary but reported an incorrect final output in the subsequent step. These issues could occur due to prompt sensitivity, multistep error propagation, or training data bias. We mitigated the errors through prompt refinements and in‐context examples, consistent with prior work showing that prompt phrasing can meaningfully affect LLM performance.^27^, ^28^

Finally, hallucination risk and automation bias remain central considerations for clinical integration. Prior work suggests that hallucinations can be made less frequent using adaptation techniques such as ICL (in‐context learning), QLoRA (quantized low‐rank adaptation), and so forth.^29^ Their findings report that the adapted models could outperform humans in terms of summarization errors in clinical practice. We emphasize that human checks remain critical to ensuring the accuracy and safety of these models. LLM outputs should be treated as extracted, structured representations of documented intent that require verification rather than definitive truth. Discrepancies between the CT simulation order and consult‐note‐derived outputs should trigger closer review and clarification rather than automated action. With appropriate safeguards, clinical AI assistants have the potential to shift effort from manual information extraction toward oversight and verification, but this transition must be validated prospectively. Future work will explore alternative decoding parameters and more diverse datasets to evaluate prospective implementation, quantify workflow impacts on end users, and define best‐practice checkpoints for safe clinical use.

## 5 CONCLUSION

This study demonstrates that general‐purpose LLMs (Llama 3.3) can be adapted, solely through prompt engineering, to interpret radiation oncology consult notes and perform anatomical site identification with accuracy comparable to human experts. Beyond overall accuracy, our error analysis revealed that model errors were not random failures, but prompt design artifacts and high‐level reasoning disagreements congruent to those of human reviewers, suggesting a human‐like pattern of interpretation. These findings transform the model into a more interpretable system, demonstrating that its limitations are logical, addressable, and often tied to source documentation. The notable error overlap between human experts and the LLM suggests that the model is sensitive to the same documentation ambiguities that challenge human experts and may help identify complex cases where the LLM‐derived treatment‐site descriptions disagree with existing CT simulation documentation, prompting additional review. Our study shows that this approach can provide a solution to reducing documentation burden, standardizing data, and mitigating a tangible source of inefficiency in the clinical workflow.

## AUTHOR CONTRIBUTIONS

**Klea Hoxha**: Directed all content; technical development of the model; result analysis; manuscript writing, reviewing, and editing. **Ricky R. Savjani**: Conceptualization of study; resources; reviewed and provided feedback for the manuscript. **Jack Neylon**: Contributed to the technical development of script for data extraction and import from the clinical server. Reviewed and provided feedback for the manuscript. **Jonathan Pham**: Independent data review and annotation; reviewed and provided feedback for the manuscript. **Rojine Ariani**: Independent data review and annotation; reviewed and provided feedback for the manuscript. **Dylan P. O'Connell**: Conceptualization of study design and methodology; supervision; manuscript writing, reviewing, and editing.

## CONFLICT OF INTEREST STATEMENT

The authors declare no conflicts of interest.

## Supporting information



Supporting Information

## Data Availability

The clinical datasets analyzed during this study contain protected health information and are not publicly available due to institutional privacy regulations. The prompt templates used in this study are available in the appendix.

## APPENDIX A PROMPT TEMPLATES USED IN THE LLM PIPELINE

### A.1

**Site isolation and classification prompts**:

**Table:**

**System prompt**	“Given are doctor's notes for each patient case, the note reports information on a patient's background, disease, and the radiation treatment plan that the doctor decided on. You are a radiation therapist that takes CT images of the anatomical site that will be treated. The CT images you take will be used to plan and set up the patient for the entirety of the treatment. The act of taking CT images that are identical in set up to treatment set up is called CT simulation.”
**Prompt 1**	‘This is the doctor's note for the patient case {MRN}: {Notes}.∖ Your goal is to isolate and extract the section or sections with the most information about the upcoming radiation treatment ∖ Prioritize sections with keywords such as objective/plan/assessment/recommendation/treatment site/metastasis∖ You MUST disregard previous radiation therapy treatments, disregard medications, only report information on the anatomical site(s) that the current radiation treatment plans will be delivered at.∖ You MUST disregard radiation treatment plans that will be redirected to another doctor. For example: ‘Patient is scheduled to see Dr. X for this metastatic site’.∖ Ensure you provide all the relevant details without omitting critical treatment information.”∖
**Prompt 2**	‘Based only on what is directly in this text, which are the anatomical sites that the therapist needs to take a CT simulation of? Report only the anatomical treatment site or sites that will be included in the CT simulation with details about the location. If the doctor is reporting anatomical sites that are to be biopsied or surgically resected, you must disregard them.∖ If there is more than one anatomical site that will be imaged in the upcoming CT simulation orders, you MUST report all of these anatomical sites.∖ When the site to be treated is prostate or prostate bed, You MUST mention lymph nodes only if they are being treated. Pelvic lymph nodes include the following: common iliac, internal and external iliac, internal obturator. If only the pelvic nodes are being treated, report ‘pelvic nodes’.∖ You must report the anatomical treatment site or sites, do not use additional verbiage such as ‘specifically’ do NOT include lesion size or spread. Report ONLY the correct, selected site.”
**Prompt 3**	‘Based only on what is directly in this input, classify each treatment site that will be imaged using ONLY the following list: Brain, Cervical esophagus, Face, Hypopharynx, Larynx, Lips, Nasopharynx, Oral cavity, Orbit, Oropharynx, Parotid, Sinonasal, Skull, Spine—cervical, Thyroid, Breast, Chest wall, Clavicle, Esophagus, Lung, Mediastinum, Pleura of the lung, Ribs, Scapula, Spine—thoracic, Sternum, Supraclavicular fossa, Thymus, Adrenal gland, Gallbladder, Gastroesophageal junction, Kidney, Liver, Lower esophagus, Pancreas, Paraaortic, Retroperitoneal space, Spine—lumbar, Spleen, Stomach, Anus, Bony pelvis, Bladder, Cervix, Ovary, Prostate, Prostate bed, Pelvic nodes, Prostate and pelvic nodes, Prostate bed and pelvic nodes, Rectum, Spine—sacral, Uterus, Vagina, Vulva, Ankle, Arm, Elbow, Foot, Forearm, Hand, Knee, Leg, Shoulder, Thigh, Wrist, Total body, Bone marrow, Craniospinal, Skin, Testis.∖ Report as a single option from the provided list. For example, if the site to be imaged is femur, you must classify it as thigh. If the site to be imaged is already on the list, report it the same, for example: if the site is stomach, report Stomach; if it is prostate, report prostate.∖ If the site does not correspond to any of the categories listed, classify it in as ‘Other’ followed by the anatomical area it belongs to. For example, if the doctor is treating the pericardium but not the heart, the classification would be ‘Other thorax’.∖ If there is more than one anatomical site and they belong to different categories, report them separately.∖ Note that cervix is NOT bony pelvis and hips are categorized as bony pelvis∖ Trigeminal nerve treatments classify as Brain.∖ When the site to be treated is prostate or prostate bed, report pelvic lymph nodes in the area ONLY IF they are being treated. Pelvic lymph nodes include ONLY the following: common iliac, internal and external iliac, internal obturator.∖ If only the PELVIC node is being treated, report ‘Node only’. Note that inguinal nodes are in the groin which is classified as pelvis.∖ From the context identify and list only the anatomical sites you classified, do not use additional verbiage. *NOTE that RP nodes are NOT pelvic nodes.∖ When the treatment site is prostate, classify it as prostate. When it is Prostate and lymph nodes, it classifies as prostate and nodes. explain your choice.”
**Prompt 4**	“Based only on the sites directly in this text, further classify the sites using the following list: Head, Thorax, Abdomen, Pelvis, Extremities, Body. For example: if the previously reported site was Brain, you will classify it as Head. Report only the classifications of the sites. **Note that Lumbar spine is classified under Abdomen. Note that inguinal nodes are in the groin which is classified as pelvis.”

**AI‐generated summary prompts**:

**Table:**

**System prompt**	“You are a radiation oncologist whose goal is provide a short summary to the radiation therapists and dosimetrists that will inform them about the patient and their current radiation therapy plan.”
**Prompt 1**	‘From this physician's note: {Notes}, create a summary of the patient which includes:∖ 1. current radiation treatments: focus on the PLAN/RECOMMENDATION, Assessment sections of the note.∖ 2. the most recent diagnosis and their most recent positive testing (PSMA, FDG‐avid masses, uptake)∖ 3. Report the anatomical treatment site or sites that will be treated in the upcoming plan. Include laterality.∖ 4. Check if the patient is post‐operation (look for mentions of resection cavities, mastectomy, prostate bed, etc)∖ 5. Report any concomitant treatments∖ 6. prior radiation history∖ 7. all relevant imaging done for the upcoming treatment(s) and include dates∖ 8. all open imaging orders and include dates∖ 8. all FUTURE imaging orders and dates∖ 9. Status of implants/cardiac device is {pacemaker}. Use this unless a newer status is mentioned in the note.∖ 10. treatment planning technique mentioned in the note∖ 11. Report the fractionation scheme found in the note if it exists. ∖ 12. If patient is getting SpaceOAR report it.∖ 13. Report if patient is included in departmental trials (XXXX, etc).∖ 14. Report mobility limitations, for example: ‘patient cannot bend knees/hold arms up etc’ and if there is any indication, they are claustrophobic.∖ 15. Report ‘Neurosurgery required’ if the treatment site is one of the following: ∖ – Meningioma,∖ – Schwannoma∖ – Pituitary∖ – AVM∖ – Functional (trigem, thalamotomy) ∖”
**Prompt 2**	‘Based on what is directly in this text:∖ Remove age and gender information, remove medications EXCEPT chemotherapy, remove mentions of colonoscopy. You MUST include whether the patient is receiving any concurrent treatments∖ 0. From the anatomical site or sites you isolated, list the organs at risk around it.∖ 1.remove staging information, Gleason scores,∖ 2.remove genetic testing (Decipher, PROSTOX, etc)∖ 3.remove mention of ADT∖ 4.remove mention of specific chemo drugs but leave mention of chemo and if it is neoadjuvant∖ 5. Remove mentions of CT simulations.∖ 6. REMOVE SPACEOAR mentions for all sites except PROSTATE, PROSTATE BED, PROSTATE with Nodes, PROSTATE BED with Nodes∖ 7. Check the site and report ‘Neurosurgery required’ if the treatment site is one of the following:∖ – Meningioma,∖ – Schwannoma∖ – Pituitary∖ – AVM∖ – Functional (trigem, thalamotomy)∖ Use limited verbiage∖ Keep all mentions of open/future imaging orders∖ Always keep any references about previous radiation history∖ Here is an example of a how/what to report:∖ 'The diagnosis is HPV‐associated Squamous Cell Carcinoma of the right tonsil with involvement of the hard and soft palate and base of tongue and 1/20 involved right level 2A lymph node.∖ Anatomical sites to be treated: Right tonsil, hard and soft palate, and base of tongue∖ Relevant imaging:∖ – PET/CT (MM/DD/YYYY) ∖ – CT Abd/Pelvis wo contrast (MM/DD/YYYY)∖ Open Imaging orders:∖ – MRI/CT (MM/DD/YYYY)∖ No prior radiation history∖ No pacemaker or cardiac device∖ No reported mobility issues∖ concomitant treatments: adjuvant concurrent chemotherapy + radiation.∖ The patient is post‐operative: undergone TORS right radical tonsillectomy, partial right tongue base resection, partial right pharyngectomy, right neck dissection, and left tonsillectomy.∖
	Organs at risk: spinal cord, salivary glands, oral cavity∖ Upcoming PET/CT for radiation planning is scheduled.∖ Neuro/coplanning: None∖ Here is another example but for prostate cancer: ∖ 'The diagnosis is prostate cancer with a PSMA PET on DD/MM/YYYY showing focal radiotracer uptake in the prostate, consistent with biopsy‐proven prostate cancer, with no radiotracer avid regional or nonregional lymph nodes or distant disease.∖ Anatomical site to be treated: Prostate only, no nodes.∖ The patient will receive concatenated treatment with systemic therapy as part of a clinical trial.∖ No prior radiation history∖ Relevant imaging: ∖ – MRI Prostate on DD/MM/YYYY, ∖ – PSMA PET on DD/MM/YYYY ∖ No implants or cardiac devices∖ Treatment planning technique: MR‐guided SBRT∖ Fractionation scheme: 5 fractions∖ Patient will be getting SpaceOAR∖ Organs at risk: rectum, bladder∖ Department Trial involvement: yes, XXXX Trial∖ No mobility limitations reported∖ Neuro/coplanning: none”

## APPENDIX B LLM FAILURE MODES WHEN COMPARED TO GROUND TRUTH

### B.1

**Table:**

LLM output	Ground truth	Source text
Prostate and nodes	Prostate	No text insinuating nodal involvement
Para‐aortic nodes, Vaginal apex	Para‐aortic	“….includes a CT simulation, possible placement of fiducial marker seeds to better delineate the vaginal apex”
Prostate bed, nodes	Prostate bed	No text insinuating nodal involvement
RLL, LUL, right lung, LLL	RLL, RUL	“…Additional lesions in the right lung which are PET avid, and LLL sub‐solid nodule clinically suspicious for adeno spectrum lesion…”
Prostate, L3, sacrum	Sacrum, L3	“Since Mr. XXXX has evidence of active disease in his prostate, he may benefit from RT to the primary as well.”
Right frontal convexity, medulla	Right frontal convexity	“…enhancing dural based mass along the right frontal convexity and another extra‐axial lesion posterior to the left medulla…”
Rectal/Anal mass	Rectum and nodes	“We recommended that the patient proceed with palliative radiation to the rectal/anal mass.”
Right tonsillar pillar, right cervical node mass, right submandibular space	Right tonsil, bilateral neck	“The encounter diagnosis was Primary tonsillar squamous cell carcinoma”

## APPENDIX C LLM REASONING AND SELF‐CORRECTION EXAMPLE

### C.1

**Table:**

LLM reasoning output	To determine the precise anatomical site(s) for the upcoming radiation treatment, we need to analyze the provided note excerpt. The note mentions that the patient has a history of ovarian cancer and has recently been diagnosed with a recurrence in the para‐aortic lymph node. The Plan/ Recommendation section discusses treatment options, including external beam radiation and brachytherapy, but does not explicitly state the treatment site. However, it mentions the logistics of external beam radiation therapy, including a CT simulation and possible placement of fiducial marker seeds to better delineate the vaginal apex. This suggests that the treatment site may be related to the vaginal apex or the pelvic region. Additionally, the note mentions the discussion of brachytherapy, which includes a simulation with placement of fiducial markers prior to the simulation, followed by a series of vaginal cylinder insertions. This further supports the idea that the treatment site is in the pelvic region, specifically the vaginal apex or the para‐aortic lymph nodes. Given the context and the discussion of treatment options, it is reasonable to conclude that the radiation treatment site is likely the para‐aortic lymph nodes, which are involved in the recurrence of the ovarian cancer.
