# Supplementary material for: Automated identification of radiotherapy treatment sites from unstructured physician notes
Source: J Appl Clin Med Phys. 2026 Mar 31;27(4):e70558. doi: 10.1002/acm2.70558 (PMC13140543; doi:10.1002/acm2.70558)
Supplement: Supplementary file 1 — Supporting Information [file ACM2-27-e70558-s001.docx]

**Supplementary Materials:**

| **Category** | **Example** | **NLP Failure** |
| --- | --- | --- |
| Contextual nuance – Negation | No other evidence of metastatic disease within the abdomen.; …therefore I do not intend to treat the regional lymph nodes. | Keyword-based systems would incorrectly generate a falsely positive match due to “metastatic disease” and “treat the regional lymph nodes”.  It cannot comprehend context and will misclassify the patient’s status. |
| Atypical Grammar/ shorthand | Status post segmental mastectomy and axillary lymph node sampling **ypTXNX…** | Deterministic approaches struggle to parse shorthand such as ypTXNX as it is not a standard grammar approach. |
| Abbreviations | … able to have intercourse only with the aid of an ED medication… | Dictionary look up for “ED” is highly ambiguous (Erectile Dysfunction, Eating Disorder, Emergency Department, etc). A rule-based approach would require curated keyword data which is impractical and not scalable. |
| Temporality Reasoning | …history of metastatic melanoma involving the brain and spine as well as radiation to L1-L2 and brain, … now with new PET avid peripancreatic LN concerning for progression of metastatic disease. | Deterministic NLP models cannot reliably construct a coherent timeline due to the multiple histories present on the example. |

**Table S1.** Detailed table providing illustrative examples of complexities found in our institutional notes:

**Table S2**. Physician’s Consultation Note and Corresponding CT-simulation order showing that the consult note is text heavy while the CT-sim lacks detail.

| **Physician Consultation Note** |
| --- |
| »CHIEF COMPLAINT: Disease Progression in Right Anterior Temporal Lobe    »IDENTIFYING DATA/ONCOLOGIC HISTORY: NAME is a XX y.o. male with history of grade IV glioblastoma, IDH wild type, s/p left temporal craniotomy and gross total resection (m/d/yyyy) and s/p adjuvant radiation with concurrent temozolomide on (m/d/yyyy). He completed further adjuvant chemotherapy (temodar) x 6 cycles on (m/d/yyyy). 1st recurrence on MRI brain (m/d/yyyy) showing nodular enhancement along the anterior margin of resection cavity s/p surgery GTR of recurrent tumor on (m/d/yyyy). He has since been on CeGAT vaccine ((m/d/yyyy)), Keytruda ((m/d/yyyy)) and Avastin ((m/d/yyyy)). 2nd recurrence on MRI brain (m/d/yyyy) showing Slight interval increase in the nodular enhancement along the left occipital lobe and subtle increase in the expansile FLAIR hyperintensity in the left periatrial white matter. He underwent left temporal craniotomy for GTR on (m/d/yyyy) surg path consistent with GBM WHO grade 4. He subsequently started trametinib (m/d/yyyy)- (m/d/yyyy)discontinued due to severe rash. 3rd recurrence on MRI brain (m/d/yyyy)showing further increase in mass-like enhancement and T2/FLAIR hyperintensity, restarted Avastin (received on (m/d/yyyy)none further) and trametinib with dabrafenib (m/d/yyyy)-(m/d/yyyy)interval imaging on (m/d/yyyy)revealed continued progression. He is now s/p left temporal craniotomy and STR on (m/d/yyyy)surgical path consistent with active glioblastoma. He completed re-irradiation to recurrent disease on (m/d/yyyy) MRI brain (m/d/yyyy) concerning for tumor progression in the right temporal lobe.    »STAGE: Cancer Staging  Brain tumor (HCC/RAF)  Staging form: Brain and Spinal Cord, AJCC 8th Edition  - Pathologic stage from (m/d/yyyy) WHO Grade IV - Signed by MD on (m/d/yyyy)    ...  Subjective:    Subjective  Interval History: PATIENT was last seen in our clinic following completion of re-irradiation to the left temporal lobe completed on (m/d/yyyy)    (m/d/yyyy)MRI brain revealed increased expansile FLAIR hyperintensity of the anterior temporal lobe, concerning for tumor progression. No associated enhancement or perfusion abnormality. There was also a new small foci of enhancement along left cisternal trigeminal nerve and cerebellar vermis measuring up to 6 mm, concerning for leptomeningeal spread of disease albeit less common with glioblastoma.    He most recently visited with his medical oncologist, DR.X and his NP on (m/d/yyyy) At this time, his MRI results were discussed with him and he was recommended to f/u with radiation oncology to discuss RT to the right temporal lobe. He was recommended to continue Avastin every 3 weeks, and Keytruda will be added to his treatment regimen on (m/d/yyyy) He will be starting DcVax in PLACE on (m/d/yyyy)      Objective:    Objective  Physical Exam:  There were no vitals taken for this visit.  Pain Information (Last Filed)    Score Location Comments Edu?  0-No pain None None None          ECOG - Eastern Cooperative Oncology Group performance status: ECOG Score - 1 - Restricted in physically strenuous activity but ambulatory and able to carry out work of a light or sedentary nature, e.g., light house work, office work.    Physical Exam  Constitutional:  General: He is not in acute distress.  Eyes:  Extraocular Movements: Extraocular movements intact.  Pulmonary:  Effort: No respiratory distress.  Neurological:  Mental Status: He is alert.  Psychiatric:  Mood and Affect: Mood normal.  Behavior: Behavior normal.    Limited by nature of telemedicine visit.    Lab Review / Pathology / Radiology:    MRI brain (m/d/yyyy)    IMPRESSION:  ...  Assessment:    Assessment  PATIENT is a XX y.o. male with history of grade IV glioblastoma, IDH wild type, s/p left temporal craniotomy and gross total resection (m/d/yyyy) and s/p adjuvant radiation with concurrent temozolomide on (m/d/yyyy) He completed further adjuvant chemotherapy (temodar) x 6 cycles on (m/d/yyyy). 1st recurrence on MRI brain (m/d/yyyy) showing nodular enhancement along the anterior margin of resection cavity s/p surgery GTR of recurrent tumor on (m/d/yyyy). He has since been on CeGAT vaccine (m/d/yyyy)- (m/d/yyyy)), Keytruda (m/d/yyyy)- (m/d/yyyy)) and Avastin (m/d/yyyy)- (m/d/yyyy)). 2nd recurrence on MRI brain (m/d/yyyy) showing Slight interval increase in the nodular enhancement along the left occipital lobe and subtle increase in the expansile FLAIR hyperintensity in the left periatrial white matter. He underwent left temporal craniotomy for GTR on (m/d/yyyy), surg path consistent with GBM WHO grade 4. He subsequently started trametinib (m/d/yyyy)- (m/d/yyyy)), discontinued due to severe rash. 3rd recurrence on MRI brain (m/d/yyyy) showing further increase in mass-like enhancement and T2/FLAIR hyperintensity, restarted Avastin (received on (m/d/yyyy), none further) and trametinib with dabrafenib ((m/d/yyyy)-(m/d/yyyy)), interval imaging on (m/d/yyyy) revealed continued progression. He is now s/p left temporal craniotomy and STR on (m/d/yyyy), surgical path consistent with active glioblastoma. He completed re-irradiation to recurrent disease on (m/d/yyyy). MRI brain (m/d/yyyy) concerning for tumor progression in the right temporal lobe for which he presents for further radiotherapy considerations.    Plan/ Recommendation:    Plan  We discussed with PATIENT and his family the results of his recent imaging. We discussed the risks and benefits of radiation therapy in the context of the patient’s disease, as well as potential alternatives to radiation therapy. We discussed that there is not a good surgical option for his area of tumor progression given the likely impairment from surgical removal of his right temporal lobe. The disease within the left temporal lobe responded relatively well to both of his prior courses of radiation. We therefore recommend a **10 fraction IMRT course to the right temporal lobe.** 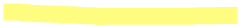   It is unclear what effect his upcoming vaccine will have on his progressive disease here. We will reassess the extent of his gross disease with an MRI once he returns from London at the end of September. If his disease has worsened, we would recommend pursuing radiation therapy. If his disease has improved, we will have another discussion about the treatment plan. He is scheduled to return on (m/d/yyyy) and so we will plan for MRI brain and CT sim to be performed that week.    We discussed the risks, benefits of radiation therapy in the context of PATIENT disease, as well as potential alternatives to radiation therapy. We explained that there may be both early and late side effects associated with radiation therapy. Acutely, these risks include fatigue, headache, nausea, seizure, and hair loss. These effects are usually self-limited and improve shortly after the end of treatment, but may persist in some patients. On a long-term basis, there is risk of edema or swelling of the target tumor (radiation necrosis) which may require treatment with steroids or, in rare cases, surgical management. We also discussed the risk of cranial nerve injury and brainstem necrosis given his multiple prior courses of radiotherapy as well, although his area of tumor progression is contralateral to the prior treated side and did not receive a clinically significant dose of radiation with the prior treatment plans. We discussed the risks, benefits, alternatives, likelihood of success, and possible results of non-treatment.    PATIENT and his family were encouraged to reach out should they have any additional questions or concerns.    PLAN:  - MRI brain and CT simulation scheduled for (m/d/yyyy)  - Plan for 10 fraction IMRT to tumor in right temporal lobe. |
| **CT Simulation Order** |
| 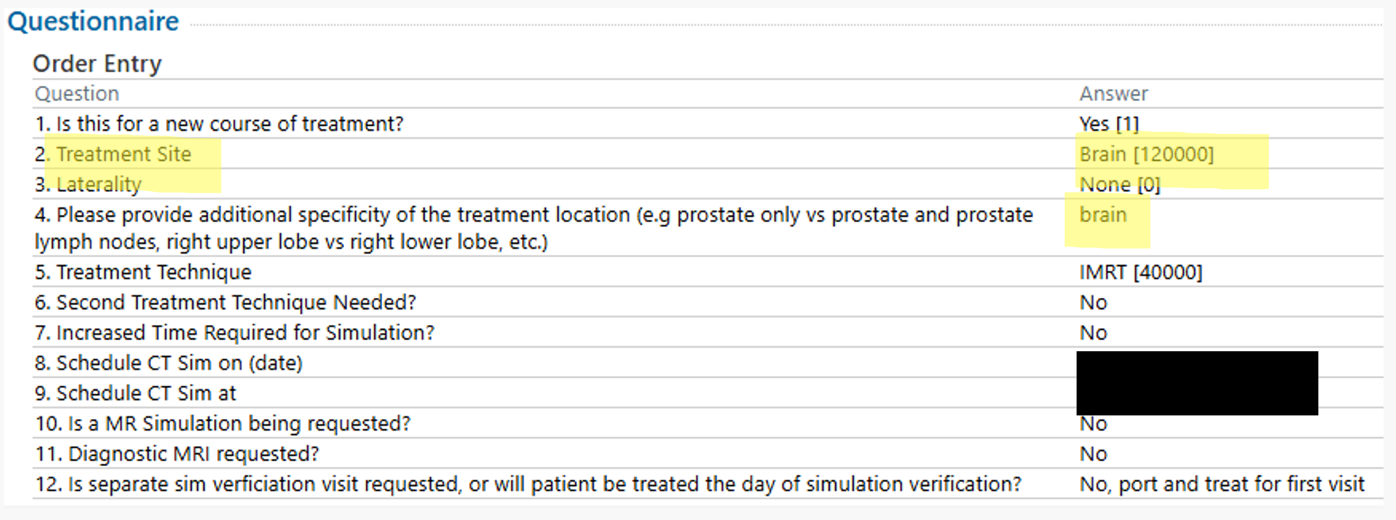 |
